# Supplementary material for: End Sequence Analysis Toolkit (ESAT) expands the extractable information from single-cell RNA-seq data
Source: Genome Res. 2016 Oct;26(10):1397–410. doi: 10.1101/gr.207902.116 (PMC5052061; doi:10.1101/gr.207902.116)
Supplement: Supplemental Material [file supp_gr.207902.116_Supp_Materials.tar.gz › ESAT-esat_v0.1/doc/html/deprecated.html]

ESAT: Deprecated List


ESAT


- Main Page
- Related Pages
- Packages
- Classes
- Files

All Classes Namespaces Files Functions Variables Enumerator Pages

Deprecated List

Member broad.core.annotation.GenomicAnnotation.getLength ()
:   Use length() instead

    Returns

Member broad.pda.seq.fastq.FastqParser.FastqParser (File fastqFile)
:   This constructure is highly discouraged as it opens a reader. Use the empty constructor instead and set the file to read, then call start.

    Parameters
    :   |  |  |
        | --- | --- |
        | fastqFile |  |

    Exceptions
    :   |  |  |
        | --- | --- |
        | IOException |  |


---

Generated on Fri Aug 15 2014 15:10:49 for ESAT by  

 1.8.7
